# Supplementary material for: Content-rich biological network constructed by mining PubMed abstracts
Source: BMC Bioinformatics. 2004 Oct 8;5:147. doi: 10.1186/1471-2105-5-147 (PMC528731; doi:10.1186/1471-2105-5-147)
Supplement: Additional File 2 — The original results of the above study (non-essential files are deleted to keep the file size under the limit set by BMC bioinformatics). [file 1471-2105-5-147-S2.bz2 › chilibotAdditionalFile2/dip10/256ID8242751E1019/html/CDKN1A_CCNA2.html]

 


 **CDKN1A** and **CCNA2** 
  
Found 163 abstracts in PubMed, retrieved 10.  
 

 What does Google say? 
 PDF only 
| .edu only 

---

**Interactive relationship** (e.g. stimulation, inhibition, etc)

**Inhibitory relationship**- p21 Waf1  [ **CDKN1A** ]  Cip1  [ **CDKN1A** ]  exerts a key role in driving this G 2 exit both by inhibiting cyclin B1 Cdk1 and cyclin A  [ **CCNA2** ]  Cdk1 2 complexes, which control G 2 M progression, and by blocking the phosphorylation of pRb family proteins.  Ref: 12881433 EMBO JEMBO J,
**Stimulatory relationship**- Thus, PD selectively elevates p21 WAF1  [ **CDKN1A** ]  CIP1  [ **CDKN1A** ]  levels and thereby arrests the cell cycle at G1 S phase by down regulating Cyclin A  [ **CCNA2** ]  Cdk2 activity.  Ref: 12819186 Carcinogenesis< MedlineTA>Carcinogenesis,
**Neutral relationship**

**Non-interactive relationship** (e.g. studied together, co-existance, homology, etc.)

- Similarly, levels of the p21 WAF1  [ **CDKN1A** ]  CIP1  [ **CDKN1A** ]  protein that is associated with the Cyclin A  [ **CCNA2** ]  Cdk2 complex increased, and these increases correlated well with the down regulation of Cyclin A  [ **CCNA2** ]  Cdk2 activity.  Ref: 12819186 Carcinogenesis< MedlineTA>Carcinogenesis,
- Panaxadiol selectively inhibits Cyclin A  [ **CCNA2** ]  associated Cdk2 activity by elevating p21WAF1 CIP1  [ **CDKN1A** ]  protein levels in mammalian cells.  Ref: 12819186 Carcinogenesis< MedlineTA>Carcinogenesis,
